# Gene Expression Changes in GABA_A_ Receptors and Cognition Following Chronic Ketamine Administration in Mice

**DOI:** 10.1371/journal.pone.0021328

**Published:** 2011-06-21

**Authors:** Sijie Tan, John A. Rudd, David T. Yew

**Affiliations:** 1 Brain Research Center, Faculty of Medicine, The Chinese University of Hong Kong, Shatin, New Territories, Hong Kong S.A.R., China; 2 School of Biomedical Sciences, Faculty of Medicine, The Chinese University of Hong Kong, Shatin, New Territories, Hong Kong S.A.R., China; The University of Hong Kong, Hong Kong

## Abstract

Ketamine is a well-known anesthetic agent and a drug of abuse. Despite its widespread use and abuse, little is known about its long-term effects on the central nervous system. The present study was designed to evaluate the effect of long-term (1- and 3-month) ketamine administration on learning and memory and associated gene expression levels in the brain. The Morris water maze was used to assess spatial memory and gene expression changes were assayed using Affymetrix Genechips; a focus on the expression of GABA_A_ receptors that mediate a tonic inhibition in the brain, was confirmed by quantitative real-time PCR and western blot. Compared with saline controls, there was a decline in learning and memory performance in the ketamine-treated mice. Genechip results showed that 110 genes were up-regulated and 136 genes were down-regulated. An ontology analysis revealed the most significant effects of ketamine were on GABA_A_ receptors. In particular, there was a significant up-regulation of both mRNA and protein levels of the alpha 5 subunit (Gabra5) of the GABA_A_ receptors in the prefrontal cortex. In conclusion, chronic exposure to ketamine impairs working memory in mice, which may be explained at least partly by up-regulation of Gabra5 subunits in the prefrontal cortex.

## Introduction

Ketamine, a derivative of phencyclidine hydrochloride (PCP), is a common anesthetic for medical and veterinary purposes. Like PCP, ketamine induces dissociative anesthesia at clinical doses [1]. Ketamine is also used as a recreational drug at nightclubs, dance parties, and rave scenes where it is commonly known as “Special K”, “Vitamin K” or “SuperK” [2]. Although ketamine is a controlled drug, its illicit use has increased rapidly in many countries and areas including the United States [3], Australia [4] and China [5].

The growing abuse of ketamine has raised concerns about its possible toxic effects. Pharmacologically, acute effects of ketamine include hypertension, tachycardia and visual alterations [6]. N-methyl-D-aspartate (NMDA) receptors are known to be intimately involved in regulating synaptic plasticity and memory function [7]. Ketamine is a non-competitive NMDA receptor antagonist, and not surprisingly causes impairments of working memory and cognitive function following acute dosing [8]–[9]. The acute effects of ketamine on memory are known, but little information is available to indicate the cognitive consequences following its long-term use [10]. Of particular concern, therefore, are results from a recent study showing hyperphosphorylation of tau in the brains of rodents and monkeys after prolonged administration [11]. Long-term ketamine administration also was shown to have detrimental proapoptotic effects on neurons, via an elevation of the Bax/Bcl-2 ratio and activation of caspase-3 [12]. Whilst the former studies indicate structural changes in the brain after chronic exposure to ketamine, it is not known if it translates to long lasting effects on cognition.

Besides blocking NMDA receptors, ketamine also binds with reasonable affinity µ-opioid receptors and sigma receptors [13], [14]. Further, anesthetic effects of ketamine probably also involve an activation of GABA_A_ receptors, which provide a major inhibitory control of neurotransmission in the central nervous system [15]. Consistent with the pharmacology of ketamine, a recent microarray study showed gene expression changes after repeated administration of ketamine in the brains of postnatal rats in various pathways linked with neurotransmission receptor signaling, such as glutamate, dopamine and GABA_A_ receptor [16]. However, gene expression changes have yet to be determined in the CNS following long-term ketamine abuse.

The prefrontal cortex (PFC), the anterior part of the frontal lobes, is regarded to be heavily involved in the central executive control of cognitive processing [17]. Alterations of interconnections among neurons in the PFC have been hypothesized to lead to a failure to integrate information with a subsequent decline of cognitive function [18]. In addition, the PFC is considered to be vulnerable to drug of abuse [19]. In the present study, we first investigated cognitive performance of mice in a long-term sub-anesthetic ketamine abuse model [11], and then checked gene expression changes in the PFC. Our results showed that increased Gabra5 was inversely related with learning and memory in long-term ketamine treated mice.

## Materials and Methods

### Animals and drug administrations

All animal experiments were approved by the Animal Experimentation Ethics Committee (AEEC) of the Chinese University of Hong Kong (CUHK) and were performed under license of the Department of Health, the Government of the Hong Kong SAR, according to the Animals (Control of Experiments) Ordinance Chapter 340(Animal License ID: (10–297) in DH/HA&P/8/2/1 Pt.13). One-month old male ICR mice were obtained from the Laboratory Animal Services Centre (CUHK), and housed at 22°–24°C with 45%–55% humidity and a 12-hour alternating light-dark cycle. Standard diet (PicoLab Rodent Diet 20, PMI Nutrition Inc., Henderson, USA) and water were available *ad libitum*. The anesthetic dose of ketamine in mice is 100 mg/kg [20], and in our previous used 30 mg/kg [11].Therefore, in the present studies, ketamine (Alfasan Inc., Utrecht, Holland) was administered intraperitoneally once per day at the sub-anesthetic dose of 30 mg/kg while the same volume of normal saline (0.9% w/v) was used as control. Body weights of the mice were measured weekly and doses adjustment appropriately. After 1 and 3 months, the mice were tested in behavioral studies and then sacrificed for subsequent gene expression analysis.

### Morris water maze

Learning and memory was assessed using a spatial acquisition task in a Morris water maze as previously described [21]. The apparatus was an open circular pool with a diameter 1.2 m filled to a depth of 0.5 M with tap water (23±1°C), which was made opaque by the addition of non-toxic black ink. The pool was divided into four equal quadrants and 4 cue cards were erected around the pool in each quadrant. A circular 12 cm diameter platform of was placed approximately 1 cm under the water surface to make it invisible to the mice. The position of the platform and the cue cards remained the same throughout the whole experiment. Mice (n = 12 from each group) were trained for 4 consecutive days, with 4 trials on each day. During each trial, individual mice were put in a randomly chosen quadrant in the pool with the head toward the pool wall. Each mouse was given 60 seconds(s) to search for and locate the submerged platform. If a mouse failed to locate the platform within 60 s, it would be guided gently to the platform. After arriving at the platform, the mouse was allowed to stay on it for 30 s. The latency time to find the platform was recorded and the average time from 4 trials represented as the daily result for the mouse. To eliminate the influence of the anesthetic effects of ketamine during behavioral studies, the tests were performed 4 hours after the last injection.

### Brain tissue collection and RNA extraction

At the end of 1 or 3 months and following behavioural testing, the mice were killed and the brains were carefully removed and washed in cold saline. The prefrontal cortex was located by reference to a mouse brain atlas [22] and dissected out immediately. For microarray and real-time PCR analysis, the tissues were soaked in RNAlater (Qiagen Inc., Valencia, CA, USA) and stored at 4°C for no more than 2 weeks. Total RNA was isolated from brain tissues using RNeasy Lipid Tissue Mini Kits (Qiagen Inc., Valencia, CA, USA) according to the manufacture's protocol. The yield of total RNA was measured at 260 nm using a Spectrophotometer. For western blot, brain tissues were rapidly frozen in liquid nitrogen and stored at −80°C until use.

### Microarray analysis

Two samples from the 3 month ketamine group and 2 from the 3 month saline group were randomly chosen for use in the Microarray analysis (n = 4). Purity and integrity of RNA samples were evaluated using a RNA 6000 LabChip kit in an Agilent 2100 Bioanalyzer (Agilent Technologies, Palo Alto,CA, USA). The samples had a high quality and integrity with A260/A280 between 1.9 and 2.1 and RIN (RNA integrity number) greater than 8.5(data not show). We used the Mouse Gene 1.0 ST GeneChip (Affymetrix Inc., Santa Clara, CA, USA) (n = 4). This array interrogates 28,853 well annotated genes offering whole-transcript coverage. The genechip process, specifically sample labeling, hybridization, stringent washing, staining and scanning of arrays, was performed at the Genome Research Centre, the University of Hong Kong. Quality control assessment of the gene expression data were performed using Affymetrix Expression Console™ software. After passing quality control, data were further analyzed for fold changes and Gene Ontology (GO) analysis using GeneSpring GX 9 software (Agilent Technologies, Palo Alto, CA, USA).

### Quantitative real-time PCR

Quantitative real-time PCR (qRT-PCR) (n = 6 per group) was performed as previously described [23], with minor modifications. Briefly, cDNA was synthesized in a 20 µl reaction volume containing 1 µl oligo(dT)_12–18_ (500 µg/ml), 5 µg total RNA, 1 µl dNTP mix(10 mM each) and 200 units Moloney murine leukaemia virus (M-MLV) Reverse Transcriptase (Invitrogen, Carlsbad, CA, USA). The amplification reactions were performed using a 7900 HT Fast Real-Time PCR System (Applied Biosystems, Foster City, CA, USA) in 96 well plates. The PCR cycle parameters were 10 min at 95°C, and 40 cycles at 95°C for 15 s and 60°C for 60 s. Total 50 µl reaction volumes included 25 µl Power SYBR® Green PCR Master Mix kit (Applied Biosystems, Foster City, CA, USA), 1 µl cDNA and 1 µl forward and reverse primers (500 µmol/l). The primers of GABA_A_ receptor alpha 1–5 subunits and endogenous β-Actin were designed using Primer Premier 5.0 (Premier Biosoft International, Palo Alto, California, USA). The primer sequences and PCR product lengths are shown in Table 1.We chose crossed-intron primers to eliminate possible genomic DNA contaminants in the amplification process. The specificity of amplification was also confirmed by melting curve analysis with one single peak at qRT-PCR reactions. Each sample was analyzed in triplicate and normalized to endogenous control. The fold-changes of gene expression between the ketamine and saline groups were calculated using 2^-Delta Delta Ct^ methodology [24].

**Table 1:** Primers for quantitative real-time PCR analysis.

Gene	Primer sequence(5′–3′)		Product length(bp)
	Forward	Reverse	
Gabra1	CCA AGT CTC CTT CTG GCT CAA CA	GGG AGG GAA TTT CTG GCA CTG AT	111
Gabra2	TTA CAG TCC AAG CCG AAT GTC CC	ACT TCT GAG GTT GTG TAA GCG TAG C	103
Gabra3	CAA GAA CCT GGG GAC TTT GTG AA	AGC CGA TCC AAG ATT CTA GTG AA	119
Gabra4	GAG ACT GGT GGA TTT TCC TAT GG	GGT CCA GGT GTA GAT CAT CTC ACT	94
Gabra5	CCC TCC TTG TCT TCT GTA TTT CC	TGA TGT TGT CAT TGG TCT CGT CT	99
β-Actin	AGG CCA ACC GTG AAA AGA TG	ACC AGA GGC ATA CAG GGA CAA	101

### Western blot

For western blots [25] , frozen brain tissues (n = 6 from each group) were thawed and homogenized in 300 µl RIPA lysis buffer supplemented with protease inhibitor cocktail (Millipore Inc., Billerica, MA, USA). The lysates were centrifuged at 14,000 g at 4°C for 30 min and the supernatants were collected and stored at −20°C until use. Protein concentration was determined by the Bio-Rad DC protein assay (Bio-Rad Laboratories Inc., Hercules, CA, USA). Each sample of 50 µg protein was separated by 10% SDS-PAGE electrophoresis using 100 Volt for 2 hours and then transferred to a PVDF membrane at 200 mA for 60 min. The membrane was blocked with 5% non-fat dry milk for 1 h at room temperature and then incubated overnight at 4°C with primary antibodies diluted as followed: GABRA1 (1∶1,000) (ab94585, Abcam, Cambridge, MA, USA), GABRA5 (1∶1,000) (AB9678, Millipore, Billerica, MA, USA), β-actin (1∶20,000) (MAB1501, Millipore, Billerica, MA,USA). On the next day, after washing 3 times with 0.05% Tween-20 and phosphate buffered saline, the membrane was incubated with the corresponding horseradish peroxidase (HRP) conjugated secondary antibody for 1 h at room temperature. Blots were then developed using an ECL Plus Kit (Millipore) on Fuji Medical X-ray film and scanned using a Bio-Rad 6500 scanner. Optical density was quantified with Quantity One software (Bio-Rad).

### Statistical analysis

Significance of differences between Morris water maze datawere assessed by repeated measures ANOVA. Results of qRT-PCR and western blot were compared by independent-sample t-test. Calculations were done using SPSS software (version 15.0). Differences were considered significant when *p*<0.05.Data are presented as mean ±SD.

## Results

### Behavioral studies

**Figure 1:**
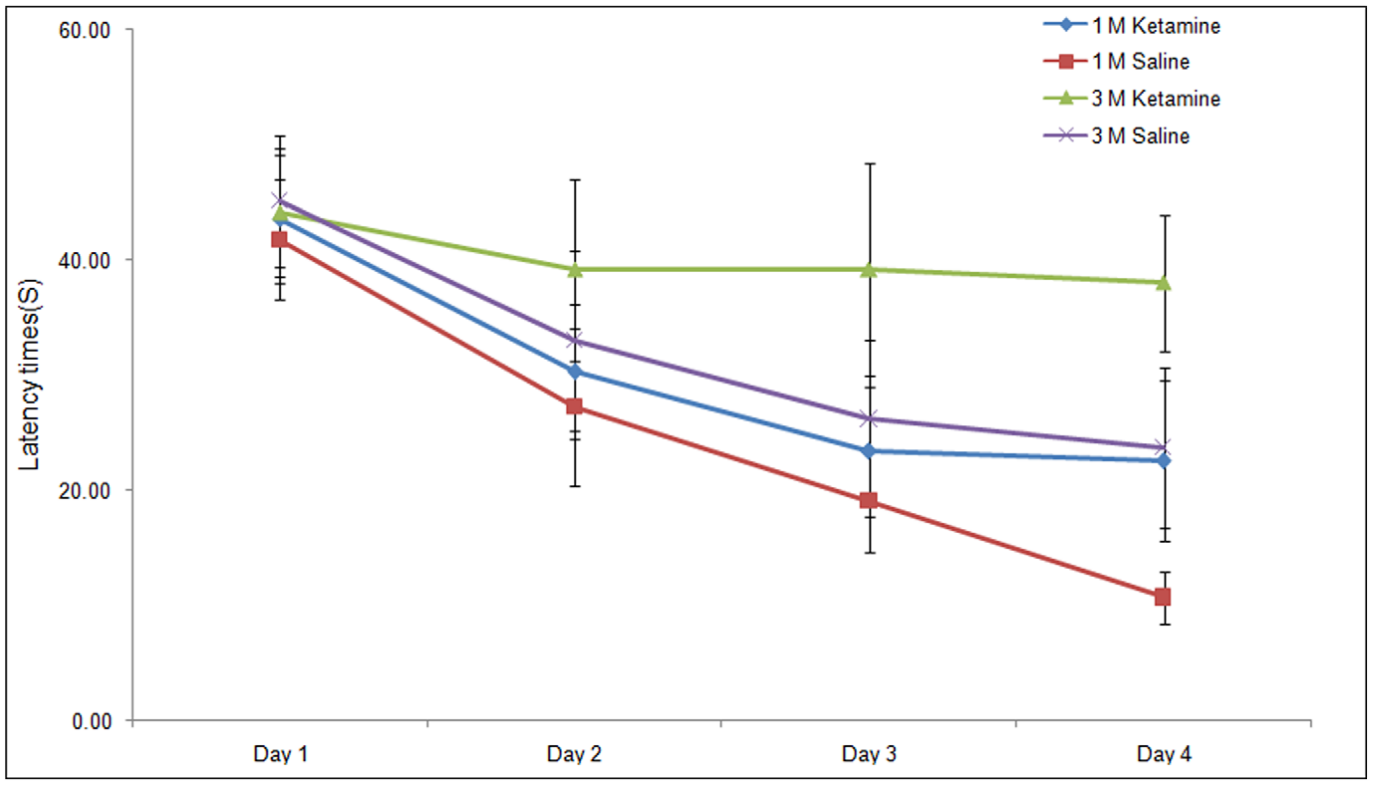
Spatial learning and memory performance of mice in the Morris water maze following 1 (1M) and 3 months (3M) treatment with ketamine. Significant differences between groups were analyzed using a repeated Measures ANOVA. 1M saline vs 1M ketamine, *p*<0.05; 3M saline vs 3M ketamine, *p*<0.05.

The Morris water maze was used to assess spatial memory. The times of the mice to find the escape platform were recorded. On day 1, there were no statistical differences between the latency times of the animals in the ketamine or control group to reach the hidden platform. However, on day 4, the ketamine treated animals needed a significantly longer time compared with the saline treated animals to find the platform (*p*<0.05). Repeated measures ANOVA revealed consistent significant effect of long-term ketamine administration on latency times (Figure 1), suggesting that there was a decline of cognitive ability compared with saline controls (*p*<0.05).

### Microarray analysis

The microarray results of this study were deposited in the NCBI Gene Expression Omnibus database (Accession Numbers: GSM647254- GSM647257). Data revealed that 110 genes were up-regulated and 136 genes were down-regulated more than 1.3 folds in the brains of the ketamine-treated mice (Table 2). These 246 genes were further explored using GO analysis. The gamma-aminobutyric acid signaling pathway (GO: 0007214) and the index of GABA_A_ receptor activity (GO: 0004890) were amongst the 12 most significantly affected systems (Table 3). The change for Gabra5 (Accession number: NM_000810) was +1.65 fold.

**Table 2:** Numbers of different expressed genes in the brain of ketamine treated mice.

Fold Change	Total Gene no.	No. up-regulated	No. down-regulated
>2.0	12	2	10
>1.9	21	3	18
>1.8	27	5	22
>1.7	37	12	25
>1.6	45	18	27
>1.5	64	25	39
>1.4	103	41	62
>1.3	246	110	136

**Table 3:** 12 most statistically significant changed terms in the Gene Ontology analysis.

GO ACCESSION	GO Term	p-value
GO:0042136	neurotransmitter biosynthetic process	0.000175264
GO:0007214	gamma-aminobutyric acid signaling pathway	0.000382054
GO:0042401	biogenic amine biosynthetic process	0.000563124
GO:0004890	GABA-A receptor activity	0.000666473
GO:0042398	amino acid derivative biosynthetic process	0.000721329
GO:0016917	GABA receptor activity	0.000837374
GO:0042133	neurotransmitter metabolic process	0.001027183
GO:0003677	DNA binding	0.001408004
GO:0008292	acetylcholine biosynthetic process	0.001535487
GO:0004753	saccharopine dehydrogenase activity	0.001535487
GO:0047131	saccharopine dehydrogenase (NAD+, L-glutamate-forming) activity	0.001535487
GO:0005307	choline:sodium symporter activity	0.001535487

### Quantitative real-time PCR

**Figure 2:**
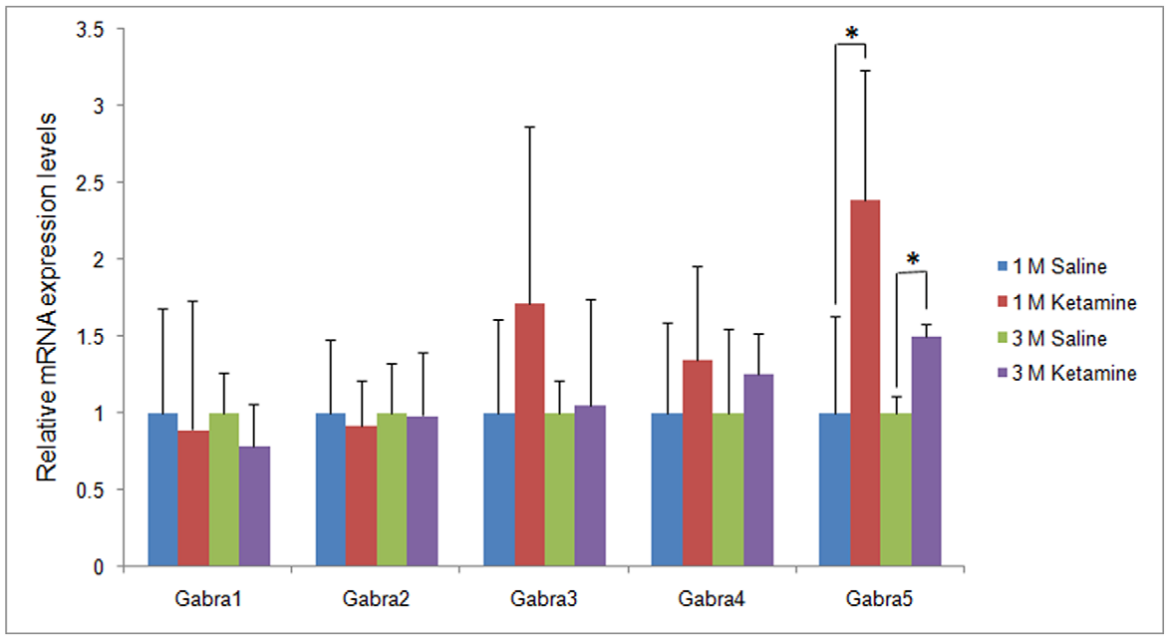
Gene expression changes of 5 GABA_A_ receptor subunits in the prefrontal cortex as reveled by quantitative real-time PCR. Significant gene expression changes were found for Gabra5 (* *p*<0.05).

To confirm the genechip results, mRNA levels of 5 GABA_A_ receptor subunits, namely Gabra1-5, were evaluated by qRT-PCR. After 1month ketamine administration, Gabra5 mRNA levels in the prefrontal cortex of the ketamine treated mice was 2.36±0.85 fold higher than levels observed in the control saline group (*p*<0.05). Similarly, significant changes of Gabra5 were also found in the animals treated for 3 months (1.4963±0.08264 fold, *p*<0.05) (Figure 2). Conversely, there was no significant difference in gene expression levels for other GABA_A_ receptor subunits following for 1 and 3 months ketamine treatment, respectively (*p*>0.05) .

### Western blot

**Figure 3:**
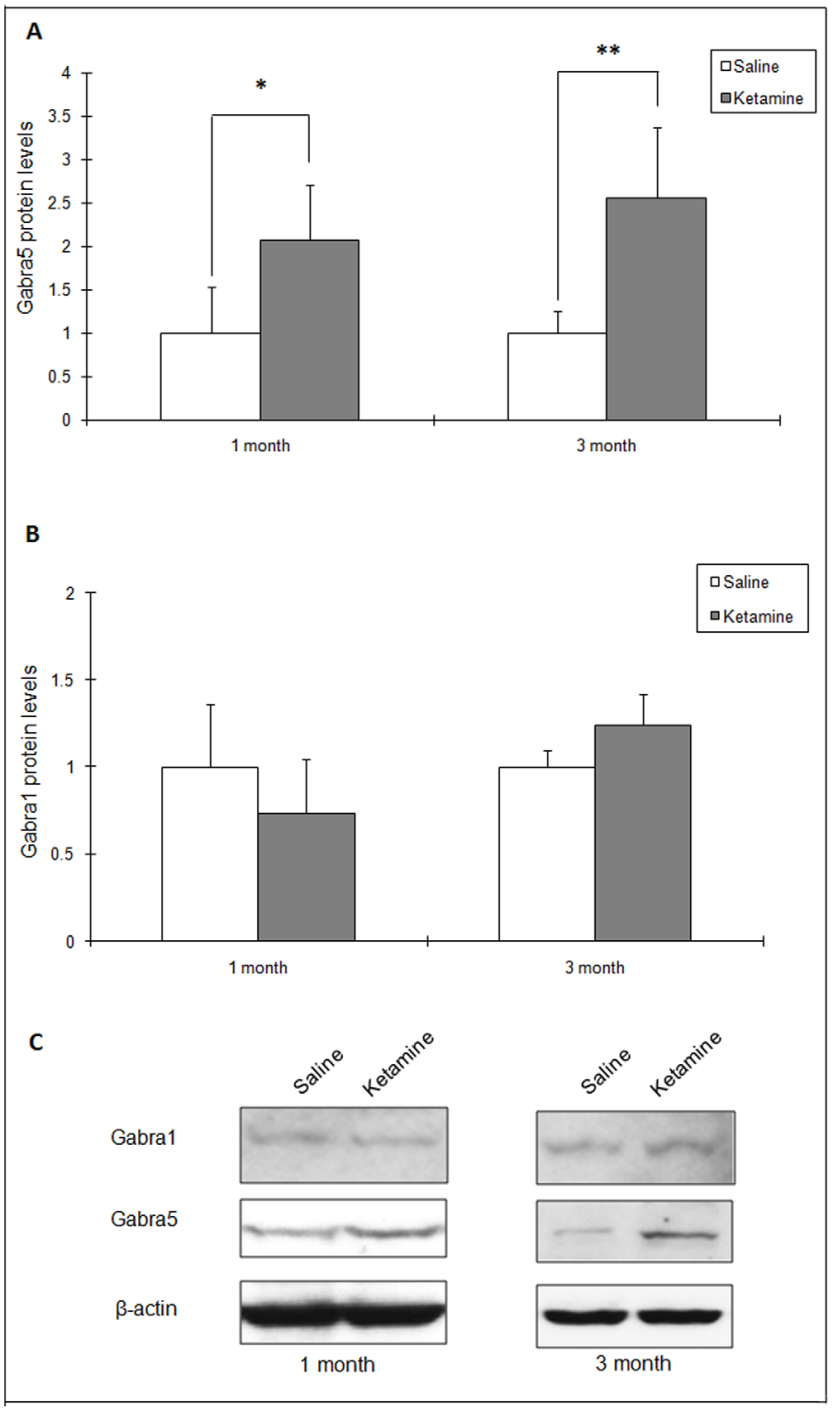
Changes of 5 GABA_A_ receptor subunits in the prefrontal cortex as reveled by Western blot. (A) Fold changes of the protein Gabra5 in the prefrontal cortex at 1- and 3-months (*p<0.05, ** *p*<0.05). (B) Fold change of the protein Gabra1 in the prefrontal cortex at 1- and 3-months. (C) Representative images of western blot results for Gabra5 and Gabra1.

Western blots were performed to investigate the protein level changes of GABA_A_ receptors in ketamine treated mice. Fold changes and representative images of western blot results are shown in Figure 3. Figure 3(A) shows western blot results of Gabra5. Gabra5 was significantly up-regulated following 1 and 3 months treatment with ketamine when compared with the respective controls (1.97±0.55 for 1 month, 2.56±0.81 for 3 months, *p*<0.05; Figure 3(A)). For Gabra1, no significant changes were found at 1 month (0.74±0.31 *p*>0.05), but at 3 months, a marginal significant difference was observed (1.24±0.17, p = 0.05, Figure 3(B)).

## Discussion

The major finding of the present study was that long-term ketamine administration impaired working memory and up-regulated the Gabra5 gene in the prefrontal cortex (PFC).These results suggest that long-term ketamine administration use may affect cognition by increasing the activity of a tonic GABA signaling system.

NMDA receptors are the major excitatory ligand-gated ion channel in CNS [26], [27]. Moreover, activation of NMDA receptors plays a vital role in mechanisms of long-term potentiation (LTP), which is believed to be a fundamental key facilitating synaptic plasticity and memory formation [7]. Thus, blocking NMDA receptors predictably disrupts LTP and impairs cognition [28], [29]. Indeed, the short-term use of ketamine has been found to impair memory in human subjects [9]. In another study, the short-term use of ketamine impacts on memory process during recruitment of frontal–parietal regions as revealed by neuroimaging [30]. In our studies, long-term injection of ketamine impaired learning and memory during its ongoing scheduled administration. Following one-month of treatment, mice on the control saline schedule found the hidden platform quickly after 3 days of training, while mice that had received ketamine spent more time to locate it. After 3 months, the latency times of mice on the ketamine schedule hardly declined (see Figure 1). It is worthy to mention that we also observed age-related memory impairments between the 1- and 3-month control animals on the saline schedule; indeed data from the 3-month animals were close to the animals that had received ketamine for 1-month. However, a more detailed analysis revealed that the trend of latency times for the 3 month saline treated animals mice improved daily, while there was no significant improvement at day 4 as compared with day 3 in the animals on the 1-month ketamine schedule. The age-related effects that we observed in the saline treated animals was predictable, since previous studies have shown that young mice aged 60 days show significantly better performance than older mice in maze learning tasks [31].

The effects of ketamine can be attributed to its ability to act as a reversible NMDA receptor antagonist and following metabolism (half life 2.5 h) the effects are transient [32]. However, the effect following long-term abuse may be more profound. In the present study, we found gene expression changes in GABA_A_ receptors as shown in the microarray results, which were also confirmed by the quantitative real-time PCR. This is consistent with evidence showing enhanced GABA_A_ receptor-mediated inhibition following ketamine injection. Thus, in guinea-pig olfactory cortical slices, ketamine has been shown to prolonging inhibition through potentiating the GABA_A_ receptor-mediated inhibitory postsynaptic currents [33]. In Xenopus oocytes expressing mouse cortical GABA_A_ receptors, ketamine enhanced inhibitory Cl^−^ currents [34]. Some indirect evidence for ketamine acting as a GABA_A_ receptor agonist is also seen *in vivo* where ketamine-induced anesthesia is partly mediated by an enhancement of central inhibitory GABA transmission [15]. As shown in our animal studies, mRNA levels of Gabra5 increased significantly after 1- and 3-month of ketamine administration. In the 3-month group, although the fold changes had decreased, significant changes were still found. Similarly, gene expression changes of Gabra5 were also confirmed by the using western blot. Gabra5 protein levels were found significantly higher in both of 1- and 3-month ketamine groups than that of their controls. Although no significant changes were found in Gabra1 levels, there were slight increases in fold changes in the 3-month group for Gabra1 as compared with 1-month group. Additionally, trends in fold changes of mRNA and protein levels were different. There may be several reasons of this discrepancy. Firstly, continuously up-regulation of mRNA would cause excessive protein synthesis, which may lead to a cumulative effect of protein levels in GABA_A_ receptors and thus the fold changes in protein levels were higher than that of mRNA. Secondly, many studies have also reported changes of GABA_A_ receptors in aging and degeneration [35], [36], [37]. An increased in GABA_A_ receptors density was thought to be the results of compensatory effects following neuronal cell loss during aging [38]. The levels of GABA_A_ receptors may also be regulated by cell signaling pathways. It was reported that serum levels of brain-derived neurotrophic factor (BDNF) were increased in chronic ketamine users [39]. Increased BDNF was also found in rat brains following prolonged ketamine exposure [40]. Elevation of BDNF probably induces a rapid increase the total number of cell surface GABA_A_ receptors through activation of Trk B receptor tyrosine kinases [41]. It would be interesting in future studies to examine whether the BDNF/TrkB/GABA_A_ signaling pathway has changed in ketamine treated mice.

GABA_A_ receptors mediate fast-acting inhibitory response in the brain and activation of GABA_A_ receptors causes hyperpolarization and decreased activity of neurons [42]. The GABA_A_ receptor system also plays a roles in memory and synaptic plasticity [43]. Compounds that enhance the action of GABA can impair memory processing, while conversely, compounds that reducing the action of GABA can enhance memory processing, especially the acquisition process [44].There are a total of a GABA_A_ receptor subunits and they have also shown differential distribution in the brain [45]. Gabra5 is predominantly expressed in brain areas important for memory processing such as level VI of cortex, and also in the hippocampus [46]. Gabra5 sets the threshold for LTP and constrains certain forms of memory through mediation of synaptic inhibition, with genetic deletion or pharmacological inhibition of the activity of Gabra5 greatly reducing the threshold [47], [48], [49]. Pharmacological blockage of GABA_A_ receptor showed treatment effects of aging associated memory decline in APP/PS1 transgenic mice [50]. Our study found that increased Gabra5 was inversely related to learning and memory following long-term ketamine treatment in mice. In particular, the learning and memory impairments were found in the 3-month ketamine treatment group, which appears in line with a recent study in human subjects [10]. Further physiological studies should focus on whether there is increased inhibitory currents via GABA_A_ receptor mediated Cl^−^ elicitation in long-term ketamine treated animals, and if such changes are reversible. Such studies would be helpful in further understanding the mechanisms of ketamine induced memory impairment.

In conclusion, we have shown that prolonged use of ketamine at a sub-anesthetic dose can impair cognitive function in mice. We also found a significant up-regulation of Gabra5 subunit in the prefrontal cortex of the ketamine treated animals. Thus, this study has revealed, for the first time, that long-term of ketamine administration and associated memory impairments may be related to an up-regulation of Gabra5 subunits in the prefrontal cortex.

## References

[1] Bergman SA (1999). Ketamine: review of its pharmacology and its use in pediatric anesthesia.. Anesth Prog.

[2] Wolff K, Winstock AR (2006). Ketamine : From medicine to misuse.. CNS Drugs.

[3] Maxwell JC (2005). Party drugs: properties, prevalence, patterns, and problems.. Subst Use Misuse.

[4] Degenhardt L, Copeland J, Dillon P (2005). Recent trends in the use of "club drugs": an Australian review.. Subst Use Misuse.

[5] Fang Y, Wang Y, Shi J, Liu Z, Lu L (2006). Recent trends in drug abuse in China.. Acta Pharmacol Sin.

[6] Ricaurte G, McCann UD (2004). Recognition and management of complications of new recreational drug use.. Lancet.

[7] Li F, Tsien JZ (2009). Memory and the NMDA Receptors.. N Engl J Med.

[8] Curran HV, Morgan C (2000). Cognitive, dissociative and psychotogenic effects of ketamine in recreational users on the night of drug use and 3 days later.. Addiction.

[9] Morgan CJ, Mofeez A, Brandner B, Bromley L, Curran HV (2004). Acute effects of ketamine on memory systems and psychotic symptoms in healthy volunteers.. Neuropsychopharmacology.

[10] Morgan CJ, Muetzelfeldt L, Curran HV (2010). Consequences of chronic ketamine self-administration upon neurocognitive function and psychological wellbeing: a 1-year longitudinal study.. Addiction.

[11] Yeung LY, Wai MSM, Fan M, Mak YT, Lam WP (2010). Hyperphosphorylated tau in the brains of mice and monkeys with long-term administration of ketamine.. Toxicol Lett.

[12] Mak YT, Lam WP, Lü L, Wong YW, Yew DT (2010). The toxic effect of ketamine on SH-SY5Y neuroblastoma cell line and human neuron.. Microsc Res Tech.

[13] Hirota K, Sikand KS, Lambert DG (1999). Interaction of ketamine with mu2 opioid receptors in SH-SY5Y human neuroblastoma cells.. J Anesth.

[14] Narita M, Yoshizawa K, Aoki K, Takagi M, Miyatake M, Suzuki T (2001). A putative sigma1 receptor antagonist NE-100 attenuates the discriminative stimulus effects of ketamine in rats.. Addict Biol.

[15] Irifune M, Sato T, Kamata Y, Nishikawa T, Dohi T (2000). Evidence for GABA(A) receptor agonistic properties of ketamine: convulsive and anesthetic behavioral models in mice.. Anesth Analg.

[16] Shi, Q, Guo L, Patterson TA, Dial S, Li Q, Sadovova N (2010). Gene expression prfiling in the developing rat brain exposed to ketamine.. Neuroscience.

[17] Baddeley, A (2003). Working memory: looking back and looking forward.. Nat Rev Neurosci.

[18] Miller EK, Cohen JD (2001). An integrative theory of prefrontal cortex function.. Annu Rev Neurosci.

[19] Perry JL, Joseph JE, Jiang Y, Zimmerman RS, Kelly TH (2010). Prefrontal cortex and drug abuse vulnerability: Translation to prevention and treatment interventions.. Brain Res Rev.

[20] Xu Q, Ming Z, Dart AM, Du XJ (2007). Optimizing dosage of ketamine and xylazine in murine echocardiography.. Clin Exp Pharmacol Physiol.

[21] Vorhees CV, Williams MT (2006). Morris water maze: procedures for assessing spatial and related forms of learning and memory.. Nat Protoc.

[22] Paxinos G, Franklin KBJ (2001). The Mouse Brain in Stereotaxic Coordinates, 2nd ed., Academic, San Diego.

[23] Li Q, Lu G, Antonio GE, Mak YT, Rudd JA (2007). The usefulness of the spontaneously hypertensive rat to model attention-deficit/hyperactivity disorder (ADHD) may be explained by the differential expression of dopamine-related genes in the brain.. Neurochem Int.

[24] Livak KJ, Schmittgen TD (2001). Analysis of relative gene expression data using real-time quantitative PCR and the 2(-Delta Delta C(T)) Method.. Methods.

[25] Wu Y, Zhang AQ, Yew DT (2005). Age related changes of various markers of astrocytes in senescence-accelerated mice hippocampus.. Neurochem Int.

[26] Cull-Candy S, Brickley S, Farrant M (2001). NMDA receptor subunits: diversity, development and disease.. Curr Opin Neurobiol.

[27] Dingledine R, Borges K, Bowie D, Traynelis SF (1999). The glutamate receptor ion channels.. Pharmacol Rev.

[28] Kleinschmidt A, Bear MF, Singer W (1987). Blockade of "NMDA" receptors disrupts experience-dependent plasticity of kitten striate cortex.. Science.

[29] Rowland LM, Astur RS, Jung RE, Bustillo JR, Lauriello J, Yeo RA (2005). Selective cognitive impairments associated with NMDA receptor blockade in humans.. Neuropsychopharmacology.

[30] Honey GD, Honey RAE, O'Loughlin C, Sharar SR, Kumaran D (2005). Ketamine disrupts frontal and hippocampal contribution to encoding and retrieval of episodic memory: an fMRI study.. Cereb Cortex.

[31] Oliverio A, Bovet D (1966). Effects of age on maze learning and avoidance conditioning of mice.. Life Sci.

[32] Hijazi Y, Boulieu R (2002). Contribution of CYP3A4, CYP2B6, and CYP2C9 isoforms to N-demethylation of ketamine in human liver microsomes.. Drug Metab Dispos.

[33] Scholfield CN (1980). Potentiation of inhibition by general anaesthetics in neurones of the olfactory cortex in vitro.. Pflugers Arch.

[34] Lin LH, Chen LL, Zirrolli JA, Harris RA (1992). General anesthetics potentiate gamma-aminobutyric acid actions on gamma-aminobutyric acidA receptors expressed by Xenopus oocytes: lack of involvement of intracellular calcium.. J Pharmacol Exp Ther.

[35] Mhatre MC, Ticku MK (1992). Aging related alterations in GABAA receptor subunit mRNA levels in Fischer rats.. Brain Res Mol Brain Res.

[36] Mizukami K, Ikonomovic MD, Grayson DR, Rubin RT, Warde D (1997). Immunohistochemical study of GABAA receptor Beta2/3 in the hippocampal formation of aged brains with Alzheimer- related neuropathologic changes.. Exp Neurol.

[37] Rissman RA, Mishizen-Eberz AJ, Carter TL, Armstrong DM (2003). Biochemical analysis of GABAA receptor subunits a1, a5, b1, b2 in the hippocampus of patients with Alzheimer's disease neuropathology.. Neuroscience.

[38] Rissman RA, De Blas AL Armstrong DM (2007). GABA(A) receptors in aging and Alzheimer's disease.. J Neurochem.

[39] Ricci V, Martinotti G, Gelfo F, Tonioni F, Caltagirone C, Bria P (2011). Chronic ketamine use increases serum levels of brain-derived neurotrophic factor.. Psychopharmacology.

[40] Ibla JC, Hayashi H, Bajic D, Soriano SG (2009). Prolonged exposure to ketamine increases brain derived neurotrophic factor levels in developing rat brains.. Curr Drug Saf.

[41] Mizoguchi Y, Kanematsu T, Hirata M, Nabekura J (2003). A rapid increase in the total number of cell surface functional GABAA receptors induced by brain-derived neurotrophic factor in rat visual cortex.. J Biol Chem.

[42] Watanabe M, Maemura K, Kanbara K, Tamayama T, Hayasaki H (2002). GABA and GABA receptors in the central nervous system and other organs.. In Rev Cytol.

[43] Jones EG (1993). GABAergic Neurons and Their Role in Cortical Plasticity in Primates.. Cereb Cortex.

[44] Chapouthier G, Venault P (2002). GABA-A receptor complex and memory processes.. Curr Top Med Chem.

[45] Olsen W, Tobin J (1990). Molecular biology of GABAA receptors FASEB J.

[46] Wisden W, Laurie, Monyer H, Seeburg PH (1992). The Distribution of 13 GABA , Receptor Subunit mRNAs in the Rat Brain I. Telencephalon, diencephalon, mesencephalon.. J Neurosci.

[47] Chambers MS, Atack JR, Broughton HB, Collinson N, Cook S (2003). Identification of a novel, selective GABA(A) alpha5 receptor inverse agonist which enhances cognition.. J Med Chem.

[48] Collinson N, Kuenzi FM, Jarolimek W, Maubach KA, Cothliff R (2002). Enhanced Learning and Memory and Altered GABAergic Synaptic Transmission in Mice Lacking the a5 Subunit of the GABA_A_ Receptor.. J Neurosci.

[49] Martin LJ, Zurek A, MacDonald JF, Roder JC, Jackson MF (2010). Alpha5GABAA receptor activity sets the threshold for long-term potentiation and constrains hippocampus-dependent memory.. J Neurosci.

[50] Yoshiike Y, Kimura T, Yamashita S, Furudate H, Mizoroki T (2008). GABA(A) receptor-mediated acceleration of aging-associated memory decline in APP/PS1 mice and its pharmacological treatment by picrotoxin.. PloS One.

